# Quantifying the hidden costs of imperfect detection for early detection surveillance

**DOI:** 10.1098/rstb.2018.0261

**Published:** 2019-05-20

**Authors:** Alexander J. Mastin, Frank van den Bosch, Femke van den Berg, Stephen R. Parnell

**Affiliations:** 1 Ecosystems and Environment Research Centre, School of Environment and Life Sciences, University of Salford, Greater Manchester M5 4WT, UK; 2 Computational and Systems Biology, Rothamsted Research, Harpenden, Hertfordshire AL5 2JQ, UK; 3 Fera, National Agri-Food Innovation Campus, Sand Hutton, York YO41 1LZ, UK

**Keywords:** surveillance, epidemic model, invasive species, early detection, diagnosis, detection

## Abstract

The global spread of pathogens poses an increasing threat to health, ecosystems and agriculture worldwide. As early detection of new incursions is key to effective control, new diagnostic tests that can detect pathogen presence shortly after initial infection hold great potential for detection of infection in individual hosts. However, these tests may be too expensive to be implemented at the sampling intensities required for early detection of a new epidemic at the population level. To evaluate the trade-off between earlier and/or more reliable detection and higher deployment costs, we need to consider the impacts of test performance, test cost and pathogen epidemiology. Regarding test performance, the period before new infections can be first detected and the probability of detecting them are of particular importance. We propose a generic framework that can be easily used to evaluate a variety of different detection methods and identify important characteristics of the pathogen and the detection method to consider when planning early detection surveillance. We demonstrate the application of our method using the plant pathogen *Phytophthora ramorum* in the UK, and find that visual inspec-tion for this pathogen is a more cost-effective strategy for early detection surveillance than an early detection diagnostic test.

This article is part of the theme issue ‘Modelling infectious disease outbreaks in humans, animals and plants: epidemic forecasting and control’. This theme issue is linked with the earlier issue ‘Modelling infectious disease outbreaks in humans, animals and plants: approaches and important themes’.

## 1. Introduction

Increased trade, travel, transportation and tourism resulting from globalization have facilitated the establishment of non-endemic pests (including animals, plants and pathogens) in new areas [1–4]. Owing to the considerable impacts these can have on human, animal, plant and ecosystem health [5], it is of vital importance that new invasions are detected as early as possible, thereby allowing the implementation of control strategies to eliminate the pest before it becomes unmanageable [6]. However, detecting pests present at a low level in the population can require considerable surveillance resources. This problem is further compounded when the pest is not easily detectable at an early stage in the establishment process. In particular, the inability of visual inspection to detect infection during the ‘presymptomatic’ period prior to the development of visible disease makes early detection more challenging [7], even when the probability of correctly detecting infected hosts (the ‘diagnostic sensitivity’) and the intensity of surveillance are high. Despite this, visual detection remains the cornerstone of early detection surveillance for emerging plant and animal pathogens. Indeed, within the UK, foot and mouth disease [8], bluetongue [9], chalara dieback [10] and ramorum disease [11–13] were all first found by visual detection.

**Figure 1.**
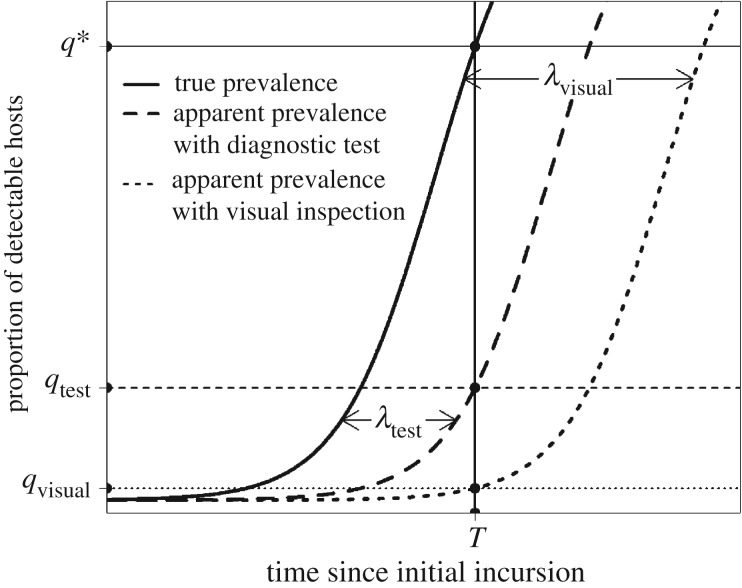
Effect of different detection lag periods on the apparent prevalence (proportion of detectable hosts) at the time of first detection. Deterministic logistic growth in the true prevalence of infection (proportion of infected individuals) over time is shown as the solid line, and the ‘apparent prevalences’ for two detection methods (a diagnostic test and visual inspection) with different detection lag periods (*λ*) are shown as dashed lines. Assuming we are using visual inspection for early detection and we detect infection for the first time at time *T*, the apparent prevalence would be *q*_visual_. However, owing to the detection lag, the true prevalence is much higher—at q*. In order to detect at a true prevalence equal to *q*_visual_, the sampling effort (and therefore the cost) would have to be greatly increased. When using a diagnostic test with a shorter detection lag (*λ*_test_), the apparent prevalence at time *T* (*q*_test_) is higher, which can be achieved with a lower sampling effort.

Early detection surveillance schemes need to be biologically, statistically and economically informed in order to be effective, yet many statistical approaches fail to account for the dynamics of pathogen spread [14]. Our previous work has shown that the proportion of infected hosts (the ‘prevalence’ of infection) at the time of first detection can be estimated by accounting for the exponential growth rate of the pathogen (*r*) as well as the rate of sampling [15,16]. We have also demonstrated how the prevalence at first detection is impacted when there is a time delay before infection is first detectable (which we term the ‘detection lag’). Figure 1 shows the change in the ‘apparent prevalence’ (i.e. the proportion of detectable hosts) over time for two different detection methods with different detection lags. A detection lag shifts the growth curve to the right by *λ* days—meaning that the apparent prevalence for any given true prevalence (e.g. the prevalence at time *T* in figure 1) will decrease as the detection lag is increased. Since this means that infection is harder to detect, the required sampling effort to detect infection at this point, and therefore the overall sampling cost, will increase. In response to issues such as this, there has been a particular focus in recent years on the development of new molecular diagnostic tests that can detect infection in the host at an early stage. These tests have been considered key to outbreak preparedness [17], but their superior test performance characteristics come with financial costs associated with test purchase or development.

The total cost of an early detection surveillance scheme (which is a key consideration owing to the long durations over which these schemes must be maintained) is therefore impacted by both the costs of applying the detection method to individual hosts and the increased sampling effort required for detection methods with longer detection lags (such as visual inspection). The threshold at which the cost of a high sampling effort outweighs the cost of a more expensive test capable of earlier detection is influenced by how quickly the pathogen is expected to spread in the population. Despite the importance of this issue to the selection of appropriate detection methods for early detection surveillance, we currently have no method of quantifying this trade-off. In the current article, we develop a novel, generic method to address this deficiency and demonstrate its application by quantifying the costs of using molecular tests instead of visual inspection for detection of the oomycete *Phytophthora ramorum* on rhododendron in the UK.

## 2. Methods

### (a) Developing a generic rule of thumb

Our previous work has demonstrated how to integrate epidemiological characteristics of a pathogen with both characteristics of the detection methods used and the statistical and financial considerations associated with sampling itself. The reader is directed to Parnell *et al.* [15,16], Alonso-Chavez *et al.* [18] and Mastin *et al.* [19] for further information. Our current method rests upon the same assumptions as this earlier work—namely, that a surveillance programme is already in place, in which *N* samples are collected every Δ days, using a detection method with a sensitivity of Se. Given that Δ is sufficiently small for us to approximate sampling as a continuous process, the rate of collection of test positive samples at time *t* (assuming a perfect diagnostic specificity) will be (Se(*N*/Δ)*q*_*t*_), where *q*_*t*_ is the prevalence at this time. In the early stages of invasion, we can assume that the prevalence is growing exponentially at a rate of *r* new infections per infection per day, but in the presence of a detection lag (which we denote as *λ*) the ‘detectable prevalence’ will be lower than this. Our previous work has demonstrated that in these cases the prevalence at first detection follows an exponential distribution, from which we can estimate any desired cumulative percentile (*q*^*x*^) as follows:

2.1 $$q^{x}=-\ln\;\left(1-\left(\frac{x}{100}\right)\right)\left(\frac{{\mathrm{re}}^{r\lambda }}{\mathrm{Se}\cdot (N/\Delta )}\right).$$

The above equation is therefore useful for situations in which we may be interested in specifying an ‘acceptable upper bound’ of the prevalence at first detection (*q*^*x*^), with (*x*/100) being an acceptable probability of reaching this prevalence or less at the time of first detection. This is also the conceptual basis of many ‘absence sampling’ programmes—in which case the aim is to demonstrate (with some degree of confidence) that, if present, the prevalence of a pathogen is lower than a given threshold.

We can also reformulate equation (2.1) to estimate the sampling effort required to be *x*% confident of detecting infection by some fixed prevalence when using a particular detection method. Multiplying this with the per-sample ‘cost’ of using the chosen detection method (*c*_method_), which includes both the cost of visiting the host and the cost of using the method, will give estimates of the total (variable) cost (*C*_assess_) of detecting infection by *q*^*x*^ using that method. This gives us *C*_assess_ = (*N*_method_/Δ_method_)*c*_method_ where (*N*_method_/Δ_method_) is the rate of sampling when using the detection method under consideration. Rearranging equation (2.1) and substituting the new total cost formulation gives

2.2 $$C_{\mathrm{assess}}=-\ln\;\left(1-\left(\frac{x}{100}\right)\right)\left(\frac{{\mathrm{re}}^{r\lambda_{\mathrm{method}}}}{q^{x}{\mathrm{Se}}_{\mathrm{method}}}\right)c_{\mathrm{method}}.$$

We can use equation (2.2) to quantify the relative performance of two different detection methods (each of which may have different values of Se, *λ* and *c*_method_) by taking the ratio of the total costs. After simplification, we obtain the following:

2.3 $$\frac{C_{{\mathrm{assess}}_{1}}}{C_{{\mathrm{assess}}_{2}}}=\left(\frac{\left(c_{{\mathrm{method}}_{1}}/{\mathrm{Se}}_{1}\right)}{\left(c_{{\mathrm{method}}_{2}}/{\mathrm{Se}}_{2}\right)}\right)\;{\text{e}}^{-r(\lambda_{2}-\lambda_{1})}.$$

As well as indicating the relative costs of the two detection methods for early detection surveillance, we can also consider equation (2.3) as a threshold. At the equivalence point (where either test would result in the same sampling costs: i.e. $(C_{{\mathrm{assess}}_{1}}/C_{{\mathrm{assess}}_{2}})=1$), the following holds:

2.4 $$\left(\frac{\left(c_{{\mathrm{method}}_{1}}/{\mathrm{Se}}_{1}\right)}{\left(c_{{\mathrm{method}}_{2}}/{\mathrm{Se}}_{2}\right)}\right)=\;{\text{e}}^{r(\lambda_{2}-\lambda_{1})}.$$

The left side of equation (2.4) is the relative expected sampling cost required for one positive detection when sampling from positive cases for method 1 compared to method 2, and the right side represents the relative increase in prevalence over the detection lag period of method 2 compared to that of method 1. If the term on the right of equation (2.4) is greater than that on the left, the most economically viable option would be to select detection method 1. If the contrary is true, detection method 2 should be selected.

### (b) The *Phytophthora ramorum* case study

We demonstrate how to estimate the cost ratio in equation (2.3) using data on *P. ramorum* in the UK, which typically affects woody ornamental shrubs (such as rhododendron) and larch trees—with the former playing a large role in spread and the latter being of particular economic and ecological importance. We have selected this pathogen because of the availability of data on its spread and detection rather than it being a prime candidate for early detection surveillance in the UK, where it is no longer considered eradicable [20] (although early detection and eradication in sub-regions are still relevant).

In response to the emergence of *P. ramorum* as an important plant pathogen in the UK, a surveillance strategy was instigated [21], conducted by trained inspection teams and based on the use of visual inspection and/or lateral flow devices (LFDs). LFDs are portable, easy-to-use, immunochromatographic tests that can be applied in the field, making them potentially useful for early detection surveillance. Although *Phytophthora* genus-specific LFDs are currently used for rapid confirmation of suspicious lesions detected by visual inspection, in the current study we consider their value as a replacement for visual detection (i.e. applied to randomly selected shrubs regardless of symptoms). We consider only surveillance of rhododendron, in which symptoms such as leaf necrosis are most apparent [22], and assume that the diagnostic specificity for detection of *P. ramorum* will be perfect, since all suspected positive samples will undergo laboratory confirmation.

The parameter estimates used in the current model are shown in table 1. We estimated the exponential growth rate of *P. ramorum* in rhododendron as the mean of the range of 0.001–0.005 shrubs per infected shrub per day reported in a recent paper [18]. A study of natural transmission of *P. ramorum* in rhododendron found a high level of symptom expression after 14 days [23], which we took as a plausible upper bound for the presymptomatic period (and therefore the detection lag for visual detection). We estimated the detection lag of the LFD as 3 days, based upon a study of detection of *P. ramorum* on rhododendon leaves using PCR and culture [24], and a study of LFD detection of the pathogen *Botrytis cinera* [25]. We used data from a proficiency test of 16 plant health inspectors for detection of *ramorum* and other *Phytopthora* diseases in rhododendron (Defra project PH0439: ‘Improving tools and approaches for Plant Health Inspectorate activities detection, surveillance and monitoring’) to estimate the sensitivity of visual inspection. Since these individuals were not necessarily specialists on *P. ramorum*, we assumed that a surveillance program would use the top 10 performing inspectors, and so the six lowest performing inspectors were removed from further analysis. Using isolation as a gold standard, a total of 588 correct diagnoses of suspected ramorum disease were made from the 900 positive inspector-samples (accounting for each positive sample being inspected by multiple inspectors), giving an estimated sensitivity of 0.65. The same samples were tested with a commercially available LFD (*Phytophthora* spp. ALERT-LF^TM^; Neogen Corporation, UK), for which 39 of the 73 positive samples were correctly identified, giving a test sensitivity estimate of 0.53.

**Table 1.** Parameter values used for the *Phytophthora ramorum* case study.

parameter	interpretation	value
*r*	epidemic growth rate	0.0033 hosts host^−1^ day^−1^
Se_1_	sensitivity of LFD	0.53
Se_2_	sensitivity of visual inspection	0.65
*λ*_1_	LFD detection lag	3 days
*λ*_2_	visual inspection detection lag	14 days
*c*_test_	cost of LFD use (visit + test)	*£*10 host^−1^+*£*6 host^−1^
*c*_visual_	cost of visual inspection (visit + inspection)	*£*10 host^−1^+*£*0 host^−1^

### (c) Method validation

Because of the difficulties in comparing the costs of detection by a specified exact prevalence in the presence of stochasticity, we evaluated the performance of our method by reformulating equation (2.3) to relate to the ratio of prevalences at first detection, assuming a fixed total cost. This ratio can be shown to be mathematically equivalent to the cost ratio for detection by some fixed prevalence in equation (2.3) by first reformulating equation (2.2) to isolate *q*^*x*^ and then taking the ratio of these prevalences. For each detection method, we simulated deterministic logistic growth in the apparent prevalence of *P. ramorum* using the parameter estimates in table 1 and starting from an apparent prevalence of 1 × 10^−8^ (based upon an estimate of the rhododendron population of the UK and in order to reduce left censoring of low prevalences at first detection). Electronic supplementary material, figure S1 shows the initial simulated growth in the true and apparent prevalences. For each total cost, we estimated the sample size per visit (*N*) as (*C*_assess_Δ/*c*_method_), assuming a sampling interval (Δ) of 28 days. We then applied the binomial theorem [15,16,18,19] to the predictions of the logistic growth model to estimate the probability of detection at each consecutive sampling point. For each total cost, we ran 100 000 realizations of a sequential sampling process, using a stochastic method (described in [19]) to determine whether each sampling resulted in detection or not—at which point, the simulation was stopped and the prevalence recorded. We then estimated the 95th percentile of these prevalences at first detection for each test and each total cost (results shown in electronic supplementary material, figure S2), as well as the ratio of these prevalences (see electronic supplementary material, figure S3). In order to capture the effect of random error in this ratio, we also randomly paired each individual simulated prevalence at first LFD detection with that for visual inspection and estimated the ratio. The median and the 95% probability interval (2.5th–97.5th percentiles) of these estimates for each total cost are shown in electronic supplementary material, figure S4.

## 3. Results

Applying the estimates in table 1 to equation (2.3), we found that the cost of using an LFD for early detection surveillance was 1.9 times higher than using visual inspection. This result was confirmed using our Monte Carlo simulation model, which found that the relative prevalence at first detection when using the LFD was consistently 1.9 times higher than that when using visual inspection, over a range of total variable sampling costs (see electronic supplementary material, figures S2 and S3). We found a similar pattern in the individual ratio estimates, with a median ratio of (1.9/1) and a 95% probability interval of around (1/20.7)–(73.0/1) (see electronic supplementary material, figure S4).

**Figure 2.**
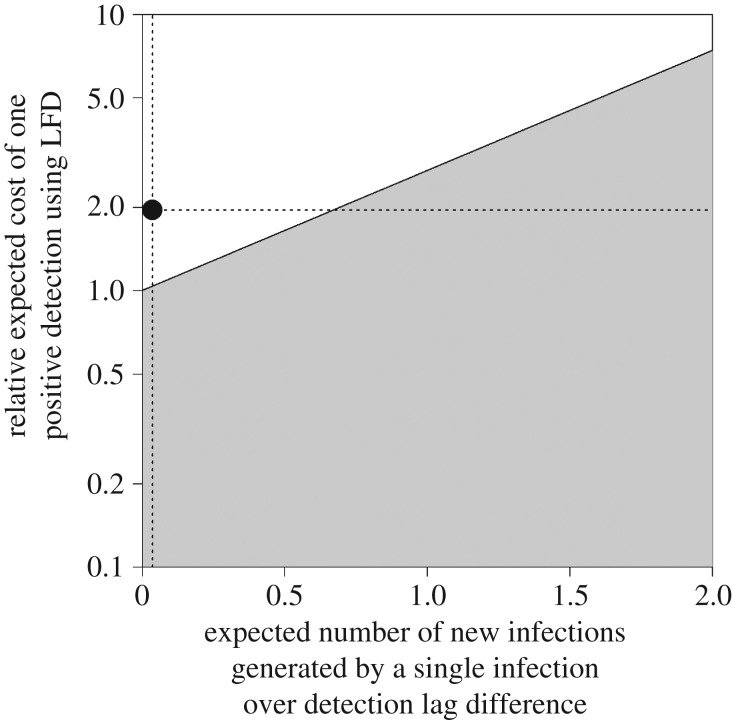
Effect of varying epidemiological and detection method parameters on the optimal detection strategy for early detection. We use the constructs in equation (2.4) as a framework, so the *x*-axis represents the terms on the right side of this equation $\left({\text{e}}^{r(\lambda_{2}-\lambda_{1})}\right)$, and the *y*-axis represents those on the left $\left(\left(c_{{\mathrm{method}}_{1}}/{\mathrm{Se}}_{1}\right)/\left(c_{{\mathrm{method}}_{2}}{\mathrm{Se}}_{2}\right)\right)$ (on a log scale, since these are ratio measurements). Higher values of *r* and/or a greater difference between the detection lag (assuming that the LFD lag is shorter than that for visual inspection) will be towards the right of the *x*-axis. On the *y*-axis, diagnostic methods with equal sensitivities and costs would be placed in the middle, with decreasing LFD sensitivity and/or higher costs moving towards the top of this axis and decreasing visual detection sensitivity and/or higher costs towards the bottom. The shaded area indicates parameter combinations giving a total cost ratio $\left(C_{{\mathrm{assess}}_{\mathrm{test}}}/C_{{\mathrm{assess}}_{\mathrm{visual}}}\right)$ of less than 1, indicating that using the LFD will minimize total costs. The unshaded area indicates where the total cost ratio is greater than 1 (where visual inspection will minimize total costs). The dotted horizontal and vertical lines indicate the values of the parameters used in the current analysis.

We also investigated the impact of parameter uncertainty on the optimal detection method for minimizing total cost, as shown in figure 2 and electronic supplementary material, figure S5. Figure 2 shows the effect of varying those parameters impacting upon the apparent prevalence curve (i.e. detection lag and exponential growth rate) on the *x*-axis, and those parameters impacting upon the cost of detecting infections (i.e. test sensitivity and detection method costs) on the *y*-axis, using the formulation described in equations (2.3) and (2.4) and in §2 above. An alternative visualization of the same results is shown in electronic supplementary material, figure S5, which shows the effect of varying individual epidemiological or detection parameters. In both cases, the parameter ranges for which inspection based upon visual inspection would be economically preferable are unshaded, and those for which the LFD should be used are shaded. Current parameters are shown as dotted lines. Assuming other parameters are fixed, the frontier between these two planes is reached with an epidemic growth rate of around 0.06; a sensitivity ratio of 1.54; a detection lag difference of 206 days; or a cost ratio of 0.85 (electronic supplementary material, figure S5).

## 4. Discussion

Recent developments in molecular biology, chemistry and immunology have resulted in the development of a wide range of new diagnostic tests that can detect infection before the development of symptoms. This information is considered highly important for mounting an effective response to epidemics [26], and therefore the potential for earlier detection has been heralded by some as the future of disease surveillance. However, these attributes come with a cost—in particular, the direct financial cost associated with their purchase—which may make them less cost-effective over the large areas and long durations required for an effective early detection surveillance system. As a result, visual detection remains the mainstay of early detection surveillance for animal and plant pathogens.

When selecting a suitable detection method for early detection surveillance, we are therefore faced with the challenge of weighing the benefits associated with the earlier and/or more reliable detection achievable with new molecular tests against the lower costs (and therefore higher achievable sampling rate) when using visual detection. In doing this, we must also account for the epidemiological characteristics of the pathogen, since the relative increase in required sampling effort (and therefore cost) for a given detection lag will be greater for faster-spreading pathogens. Despite the central importance of this issue to the sustainability of a surveillance system, there has been little attempt to date to quantify the value of these attributes for early detection surveillance. Our method addresses this deficiency whilst also linking directly with methods used for declaring the absence of a pathogen from a population.

To summarize the basis of our method, we assume a pathogen invades a new population at some unknown point in time and starts to spread. Given that we have a surveillance programme in place during this spread (collecting *N* samples every Δ days), there is an *x*% chance that the prevalence will be less than the output of equation (2.1) at the time of first detection (assuming that our detection method has a detection lag of *λ* and a diagnostic sensitivity of Se—although further work is needed to identify how to incorporate a changing diagnostic sensitivity over the detection lag period and beyond [27]). Equation (2.2) allows us to estimate the expected surveillance cost for detection by any specific prevalence using any specific detection method. This focus on a specified ‘maximum acceptable’ prevalence is the basis of most regulatory surveillance efforts for pathogens thought to be absent from an area of interest, with the threshold prevalence either prescribed by intergovernmental standard-setting organizations or determined by consideration of the impact of the pathogen and the availability of control measures. Given that an initial evaluation has been conducted and at least one detection method under consideration has been found to be economically viable for use in surveillance, we have developed a method of comparing the total surveillance costs of different detection methods (see equations (2.3) and (2.4)), which can be used to select a surveillance strategy that is cost-effective and sustainable for the necessary long periods of time. We note that our method does not currently explicitly account for other surveillance aims [28,29], such as prevalence monitoring or model parametrization.

Using data obtained from the literature on the epidemiology of European *P. ramorum* strains in rhododendron and on the performance of different detection methods, and assuming random sampling of hosts regardless of their expression of symptoms, we find that the costs of early detection of this pathogen at any prevalence are lower for visual inspection than for a commercially available LFD. Figure 2 shows that this conclusion is relatively robust to changes in parameter values, unless there are considerable increases in the exponential growth rate, the relative sensitivity of the LFD, or the absolute difference in detection lags. These changes could occur with the evolution of new strains (with faster growth rates and/or longer presymptomatic periods), or through improvements in the sensitivity of the LFD (although a perfect LFD sensitivity would only just reach the frontier in figure 2). Waiting for symptom expression before using the LFD, as is generally currently done in the field, would have constrained both the detection lag and the sensitivity of the LFD to be no greater than that for visual inspection and would therefore have resulted in a higher cost ratio.

Although we have used an example of a plant pathogen in the current report, our method can be applied to any emerging pathogen or parasite, given that sampling is an ongoing process with a reasonably short sampling interval and that the pathogen is not already established in the population. Our analysis (as demonstrated in figure 2) identifies a number of pathogen and detection method characteristics that can increase the cost-effectiveness of using a molecular detection method instead of visual detection for early detection surveillance. These are listed below, along with some examples of pathogens that may be worthy of such consideration:

(1) Fast-spreading pathogens (i.e. a high exponential growth rate), such as poliovirus, foot and mouth disease virus, or *Puccinia graminis f. sp. tritici*.
(2) Considerably earlier detection than visual inspection (as may be seen with a long presymptomatic period), such as with ebolavirus, *Leptospira interrogans*, or *Candidatus* Liberibacter spp.
(3) Higher test sensitivity than visual inspection (such as when clinical symptoms are not easily identified), for example, visceral leishmaniasis caused by *Leishmania* spp, *Mycobacterium bovis* (cervical skin test versus serological test) or cassava brown streak virus.
(4) Comparable (or lower) test cost to visual inspection, such as with *Plasmodium falciparum*, *Brucella abortus* (e.g. using the Rose Bengal test) or remote sensing for *Xylella fastidiosa* (where high coverage can be achieved at comparatively lower costs).

Exploring these other applications would be valuable, as would the application of our method to more realistic spread models and real-world data.

## Supplementary Material

Trends in apparent prevalence over time for the two detection methods.

## Supplementary Material

Prevalences at first detection for the two detection methods.

## Supplementary Material

Overall prevalence ratio for the two detection methods

## Supplementary Material

Overall prevalence ratio for the two detection methods

## Supplementary Material

Sensitivity analysis of epidemiological and detection method parameters

## Supplementary Material

Validation model

## Data accessibility

This article has no additional data.
