# Supplementary figures and images for: Quantifying the hidden costs of imperfect detection for early detection surveillance
Source: Philos Trans R Soc Lond B Biol Sci. 2019 May 20;374(1776):20180261. doi: 10.1098/rstb.2018.0261 (PMC6558562; doi:10.1098/rstb.2018.0261)

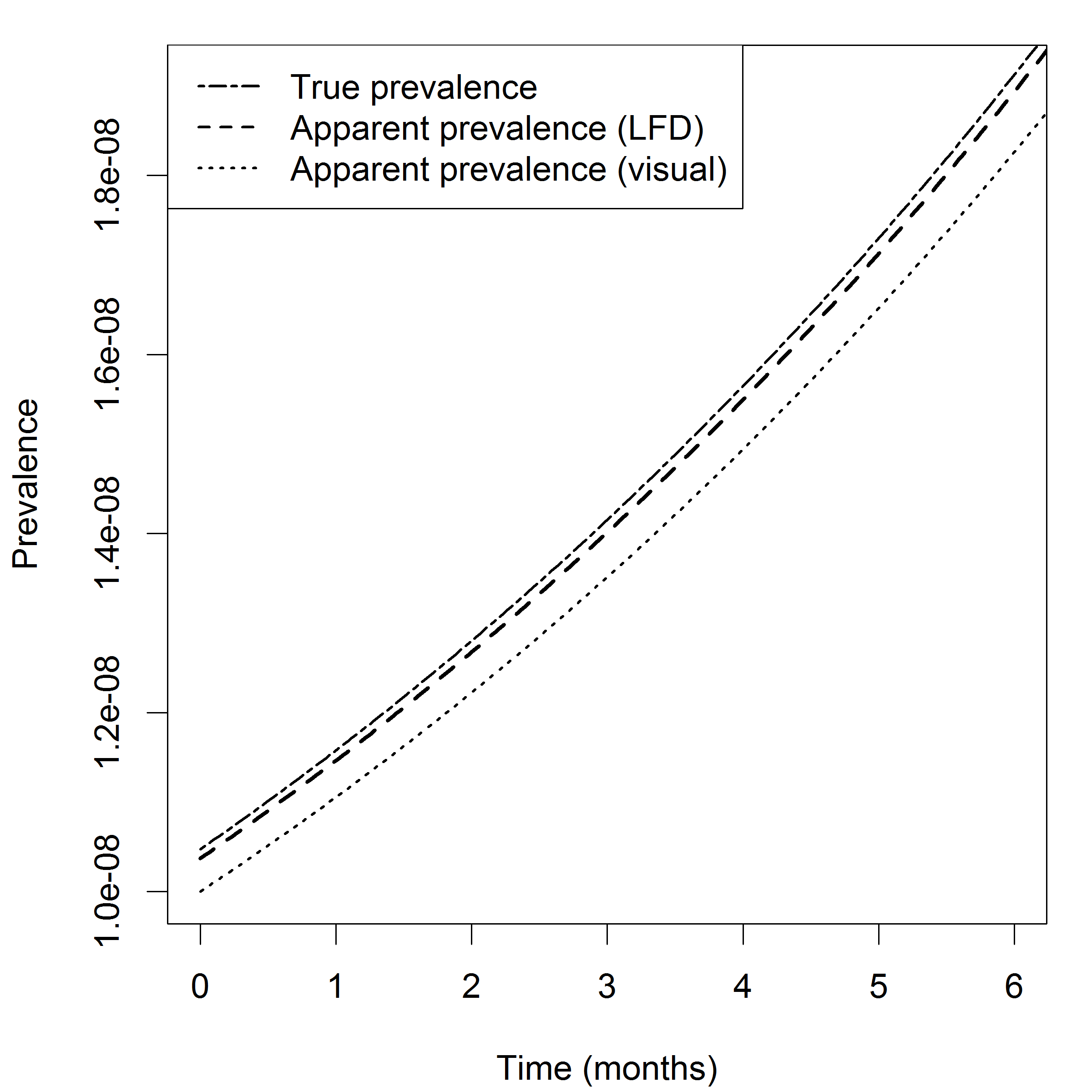

Supplement: Trends in apparent prevalence over time for the two detection methods. [file rstb20180261supp1.tiff]

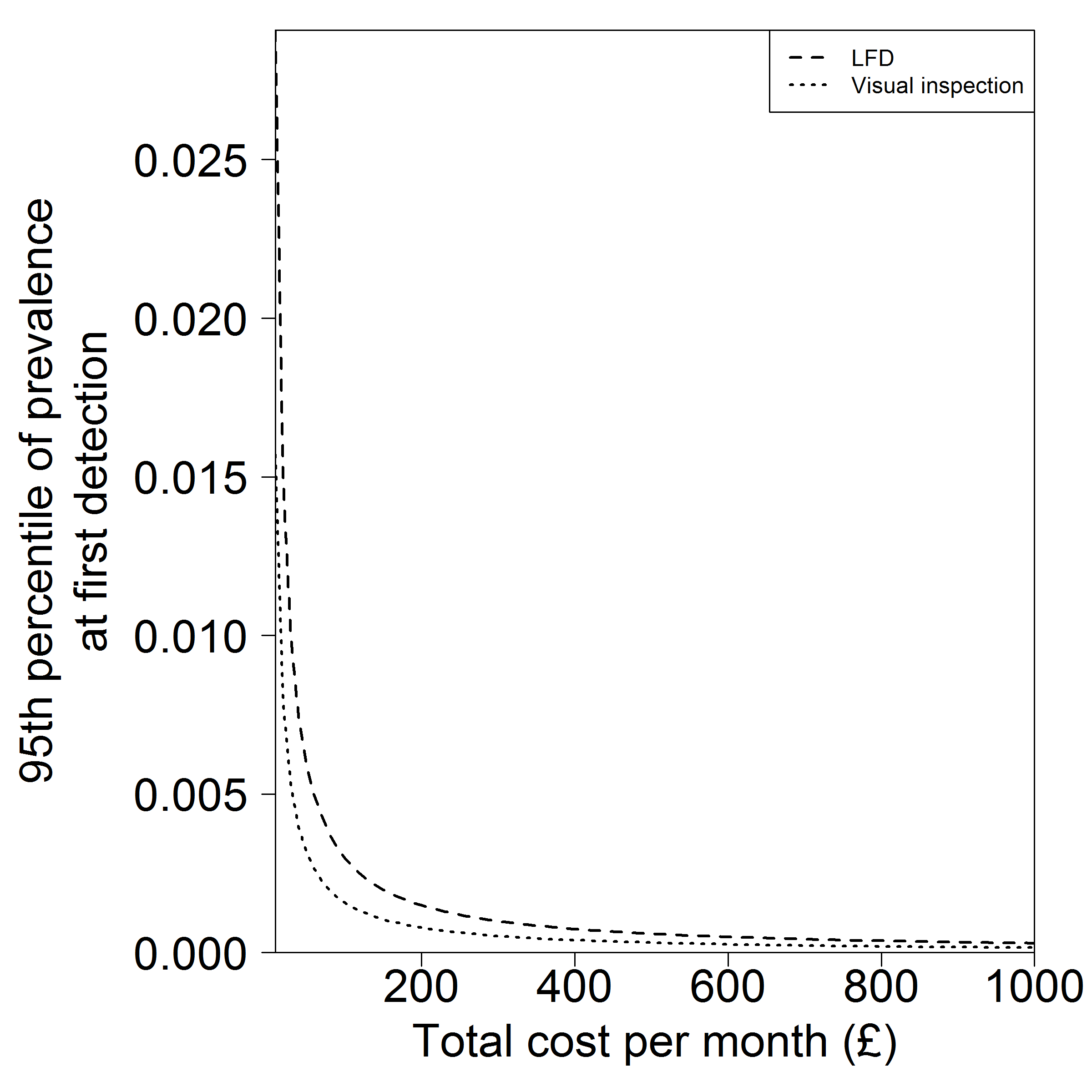

Supplement: Prevalences at first detection for the two detection methods. [file rstb20180261supp2.tiff]

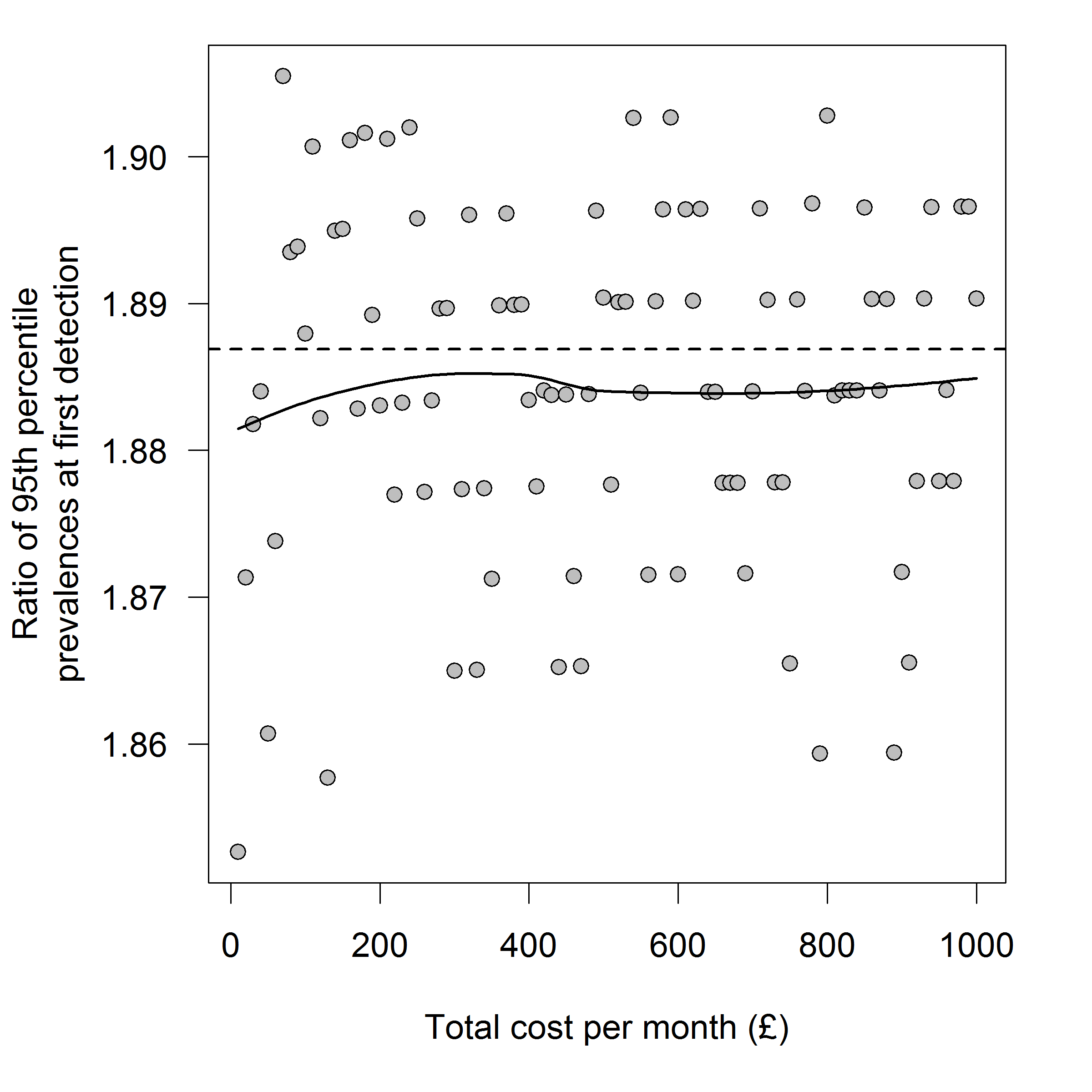

Supplement: Overall prevalence ratio for the two detection methods [file rstb20180261supp3.tiff]

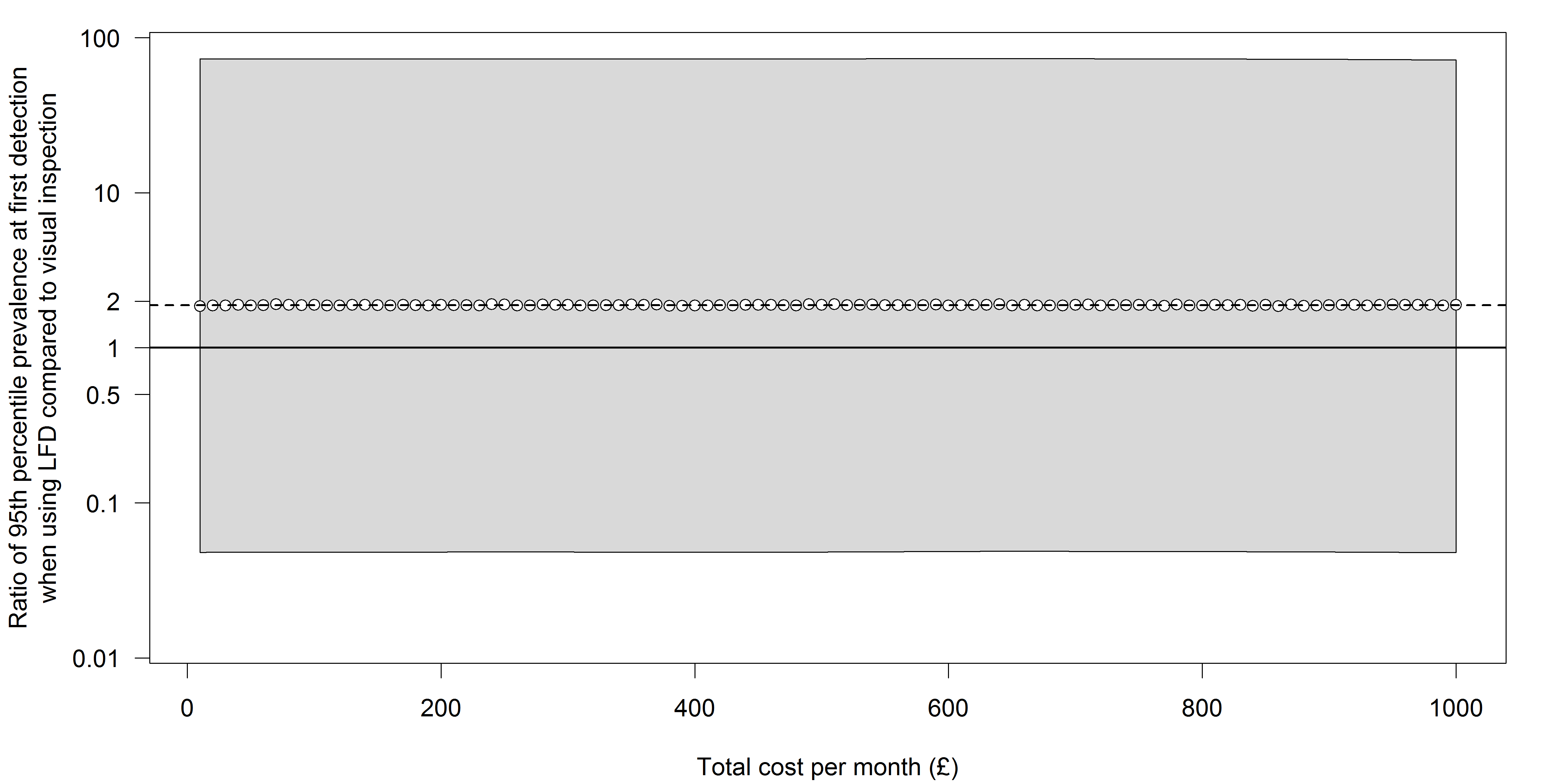

Supplement: Overall prevalence ratio for the two detection methods [file rstb20180261supp4.tiff]

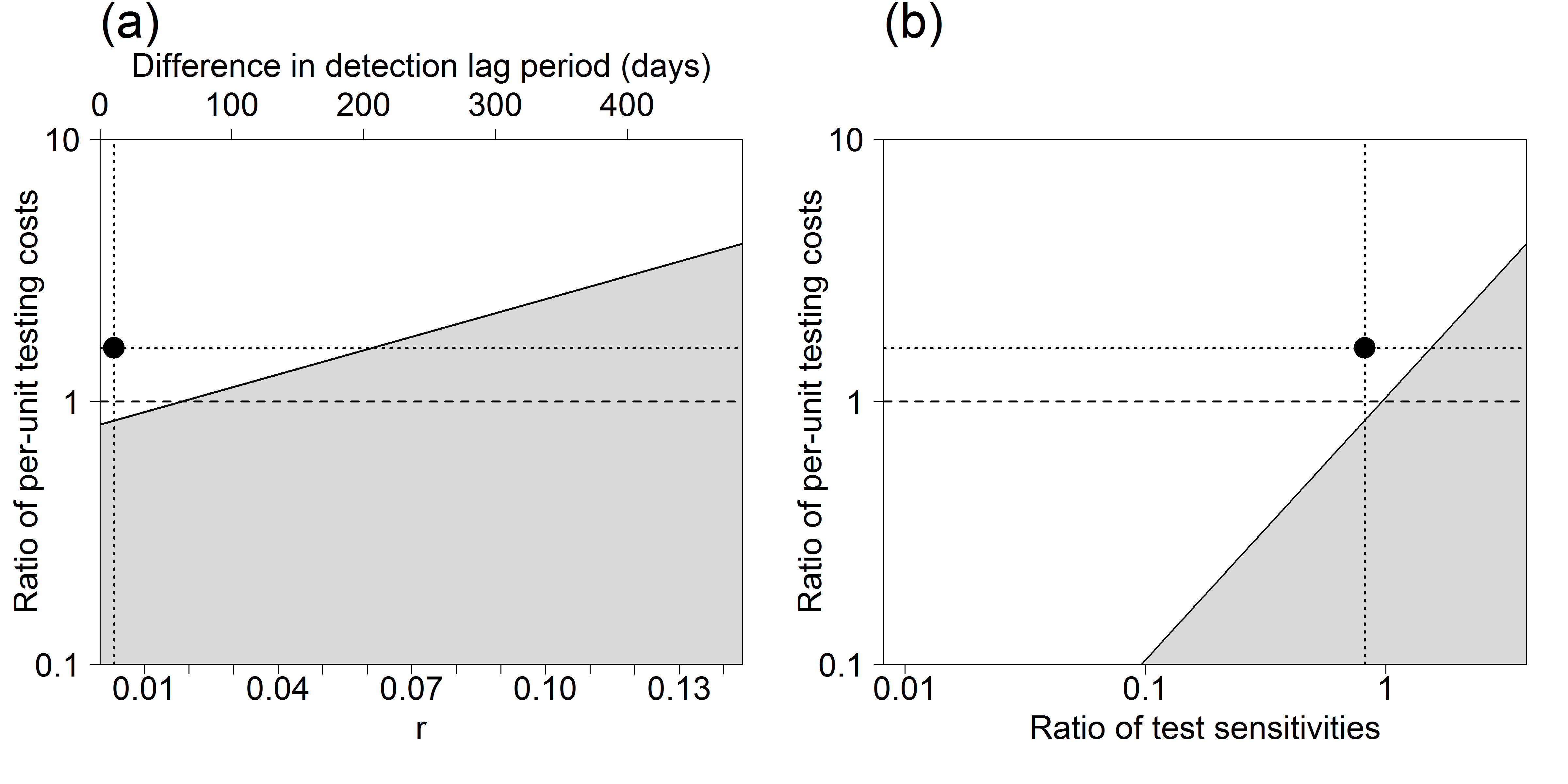

Supplement: Sensitivity analysis of epidemiological and detection method parameters [file rstb20180261supp5.tiff]
